# Novel frameshift variant in *MYL2* reveals molecular differences between dominant and recessive forms of hypertrophic cardiomyopathy

**DOI:** 10.1371/journal.pgen.1008639

**Published:** 2020-05-26

**Authors:** Sathiya N. Manivannan, Sihem Darouich, Aida Masmoudi, David Gordon, Gloria Zender, Zhe Han, Sara Fitzgerald-Butt, Peter White, Kim L. McBride, Maher Kharrat, Vidu Garg

**Affiliations:** 1 Center for Cardiovascular Research, Abigail Wexner Research Institute at Nationwide Children’s Hospital, Columbus, Ohio, United States of America; 2 Heart Center, Nationwide Children’s Hospital, Columbus, Ohio, United States of America; 3 University of Tunis El Manar, Faculty of Medicine of Tunis, LR99ES10 Laboratory of Human Genetics, Tunis, Tunisia; 4 University of Tunis El Manar, Faculty of Medicine of Tunis, Department of Embryo-Fetopathology, Maternity and Neonatology Center, Tunis, Tunisia; 5 Institute for Genomic Medicine at Nationwide Children’s Hospital, Columbus, Ohio, United States of America; 6 Department of Medicine, University of Maryland School of Medicine, Baltimore, Maryland, United States of America; 7 Department of Pediatrics, The Ohio State University, Columbus, Ohio, United States of America; 8 Department of Molecular Genetics, The Ohio State University, Columbus, Ohio, United States of America; Indiana University Purdue University at Indianapolis, UNITED STATES

## Abstract

Hypertrophic cardiomyopathy (HCM) is characterized by thickening of the ventricular muscle without dilation and is often associated with dominant pathogenic variants in cardiac sarcomeric protein genes. Here, we report a family with two infants diagnosed with infantile-onset HCM and mitral valve dysplasia that led to death before one year of age. Using exome sequencing, we discovered that one of the affected children had a homozygous frameshift variant in *Myosin light chain 2* (MYL2:NM_000432.3:c.431_432delCT: p.Pro144Argfs*57;MYL2-fs), which alters the last 20 amino acids of the protein and is predicted to impact the most C-terminal of the three EF-hand domains in MYL2. The parents are unaffected heterozygous carriers of the variant and the variant is absent in control cohorts from gnomAD. The absence of the phenotype in carriers and the infantile presentation of severe HCM is in contrast to HCM associated with dominant *MYL2* variants. Immunohistochemical analysis of the ventricular muscle of the deceased patient with the MYL2-fs variant showed a marked reduction of MYL2 expression compared to an unaffected control. *In vitro* overexpression studies further indicate that the MYL2-fs variant is actively degraded. In contrast, an HCM-associated missense variant (MYL2:p.Gly162Arg) and three other MYL2 stop-gain variants (p.E22*, p.K62*, p.E97*) that result in loss of the EF domains are stably expressed but show impaired localization. The degradation of the MYL2-fs can be rescued by inhibiting the cell’s proteasome function supporting a post-translational effect of the variant. *In vivo* rescue experiments with a *Drosophila MYL2*-homolog (*Mlc2*) knockdown model indicate that neither the MYL2-fs nor the MYL2:p.Gly162Arg variant supports normal cardiac function. The tools that we have generated provide a rapid screening platform for functional assessment of variants of unknown significance in *MYL2*. Our study supports an autosomal recessive model of inheritance for *MYL2* loss-of-function variants in infantile HCM and highlights the variant-specific molecular differences found in *MYL2*-associated cardiomyopathy.

## Author summary

We report a novel frameshift variant in *MYL2* that is associated with a severe form of infantile-onset hypertrophic cardiomyopathy. The impact of the variant is only observed in the recessive form of the disease found in the proband and not in the parents who are carriers of the variant. This contrasts with other dominant variants in *MYL2* that are associated with cardiomyopathies. We compared the stability of this variant to that of other cardiomyopathy associated *MYL2* variants and found molecular differences that correlated with disease pathology. We also show different protein domain requirements for stability and localization of MYL2 in cardiomyocytes. Furthermore, we used a fly model to demonstrate functional deficits due to the variant in the developing heart. Overall, our study shows a molecular mechanism by which loss-of-function variants in *MYL2* are recessive while missense variants are dominant. We highlight the use of exome sequencing and functional testing to assist in the diagnosis of rare forms of disease where pathogenicity of the variant is not obvious. The new tools we developed for in vitro functional study and the fly fluorescent reporter analysis will permit rapid analysis of *MYL2* variants of unknown significance.

## Introduction

Hypertrophic cardiomyopathy (HCM) is characterized by thickening of the ventricular walls in the absence of a cardiovascular or metabolic disorder that could account for the hypertrophy [1–4]. It affects 1 in 200–500 individuals and has a strong genetic component [2, 3, 5–7]. While HCM is a major cause of premature sudden cardiac death (SCD), there is significant variability in the penetrance and onset of the disease [2, 5, 8]. The majority of HCM patients are asymptomatic, while some display exercise intolerance and progressive heart failure. The ventricular chamber in HCM patients is reduced or normal in size but displays a characteristic inability to properly relax during diastole leading to progressive loss of cardiac function [3, 4, 8–10]. This progressive disease is marked by a disorganized myocyte array in the heart, with significant fibrosis of ventricular walls [7, 11–13]. Consistent with the myofibrillar disarray, about 60% of the cases of HCM have a genetic variant that is associated with the genes encoding the cardiac sarcomeric complex [4, 7, 14–16]. Of these sarcomeric genes, *MYH7*, *MYBPC3*, *TNNI3*, and *TNNT2* account for a majority of the variants [13, 16–18]. The identification of novel variants in these genes and other HCM-associated genes has increased dramatically with the advancement of high throughput genome and exome sequencing technologies [19–23]. However, the advancement of variant identification has also increased the number of potential sequence variants that could contribute to the disease in each individual, confounding the ability to assign pathogenicity to an individual variant [24–26]. While computational methods can predict the damaging effect of a variant and assist in prioritizing variants [27–32], the current consensus on the determination of pathogenicity is dependent on the identification of multiple patients showing similar symptoms harboring variants in the same gene each with strong evidence of functional impact. Functional testing is therefore critical for disorders that are associated with rare variants to better define the mechanistic link between the rare variant and the disorder [25, 33, 34].

Variants in the gene, *MYL2*, are associated with <5% of cases of HCM [5, 35]. *MYL2* encodes the *Myosin regulatory light chain*, which is expressed in the ventricular muscle and slow-twitch skeletal muscles [36]. Even though *MYL2* is considered as a candidate gene for HCM and some pathogenic missense variants have been identified and functionally tested in a few familial cases [23, 37–42], the lack of universal functional testing has prevented the designation of pathogenicity in other cases [43]. Additionally, the variable penetrance and onset of the HCM phenotype confound the ability to define the disease contribution of heterozygous *MYL2* loss-of-function variants to HCM.

Human *MYL2*, together with the essential light chain (encoded by *MYL3*), stabilizes the ‘lever arm’ of the Myosin head [44]. Human MYL2 is an 18.8 kDa protein with three major domains: a single Ca^2+^-binding EF-Hand domain at the N-terminus and two EF-Hand like domains in the C-terminus. The N-terminal region also carries a Serine residue (Ser15) which is phosphorylated by Myosin light chain kinase (MLCK) in response to Ca^2+^-mediated activation. This phosphorylation, in turn, modulates the Ca^2+^-Tropomyosin-Troponin dependent activation of Myosin motors in skeletal and cardiac muscles [36]. The phosphorylation of MYL2 by MLCK increases the interaction of Myosin head with the thin filament during each contraction cycle. This may be due to the increase in the number of cross-bridges between the myosin head and the actin filament and/or through the shift in the average position of the myosin head away from the thick filament and towards the thin filament [36, 45–49]. Correspondingly, variants in the N-terminal EF-hand domain of MYL2 that have defects in Ca^2+^ binding or MLCK phosphorylation are associated with HCM [36, 47]. Several other missense variants that have been identified in both familial and sporadic cases of HCM have been tested in vitro and using animal models [48, 50–55].

In mice and zebrafish, loss of the ventricular isoform of the regulatory light chain (*Mlc-2v* in mice and *Myl7* in zebrafish) leads to embryonic lethality with defects in ventricular sarcomere assembly. This indicates the necessity of the regulatory light chain in heart development [56, 57]. On the other hand, transgenic mice with *Mlc-2v* phosphorylation mutations develop to adulthood but display biatrial dilation, dilated right ventricle and a hypertrophic response at the molecular level [58]. This suggests that in the mouse, *Mlc-2v* point mutations are not complete loss-of-function alleles. In keeping with this observation, homozygous phosphorylation mutants in *Drosophila* regulatory light chain (*Mlc2*) display flight performance defects in adulthood while homozygous null alleles show embryonic lethality [59]. It is worth noting that in mice, heterozygous loss of ventricular regulatory light chain (RLC) does not change the protein level or cardiac function, suggesting that compensatory mechanisms may exist that maintain the level of RLC in these mice [36, 56]. Transgenic mice overexpressing an HCM-associated missense variant *MYL2*:p.E22K showed an enlarged interventricular septum and papillary muscle but failed to display sarcomeric disarray or echocardiographic changes in myocardial thickness or function [60], while transgenic mice overexpressing the DCM variant MYL2:p.D94A display dilation of the left ventricular chamber, decreased ejection fraction and mild ventricular systolic dysfunction similar to that observed in patient with the same variant [53, 61].

Such a discrepancy in mouse models carrying human variants suggests that disease pathogenesis may differ based on the specific *MYL2* variant. A report of recessive *MYL2* pathogenic variants has been reported in a Dutch family, as well as in an Italian patient with skeletal muscle Fiber-type I hypotrophy along with cardiomyopathy, further adds to the variability in disease manifestation [62]. In these cases, *MYL2* variants are thought to be loss-of-function variants, affecting individuals as homozygous recessive or compound heterozygous variants [62]. Heterozygous carriers of the variant were reported to be asymptomatic mirroring observations in mouse models, where the heterozygous loss of *Mlc-2v* does not cause a functional defect [56, 62].

In this study, we report a novel recessive variant in *MYL2* identified through exome sequencing in a family where multiple infants have died within one year of age and two of whom were diagnosed to have HCM and mitral valve dysplasia. The parents, who are heterozygous carriers of the variant, had a normal echocardiographic evaluation. Using *in vitro* analyses, we demonstrate that the mutant protein harboring this variant is not stable, and using *in vivo* functional analyses, we show that this variant is a loss-of-function allele. By comparing this variant to other HCM-associated variants, we propose a role for the C-terminal EF-hand domain in determining the localization of the protein. This work will inform the evaluation of new variants in *MYL2* and provide tools to facilitate the rapid screening of these variants.

## Results

### Family with multiple infantile-onset hypertrophic cardiomyopathy and premature death

**Fig 1:**
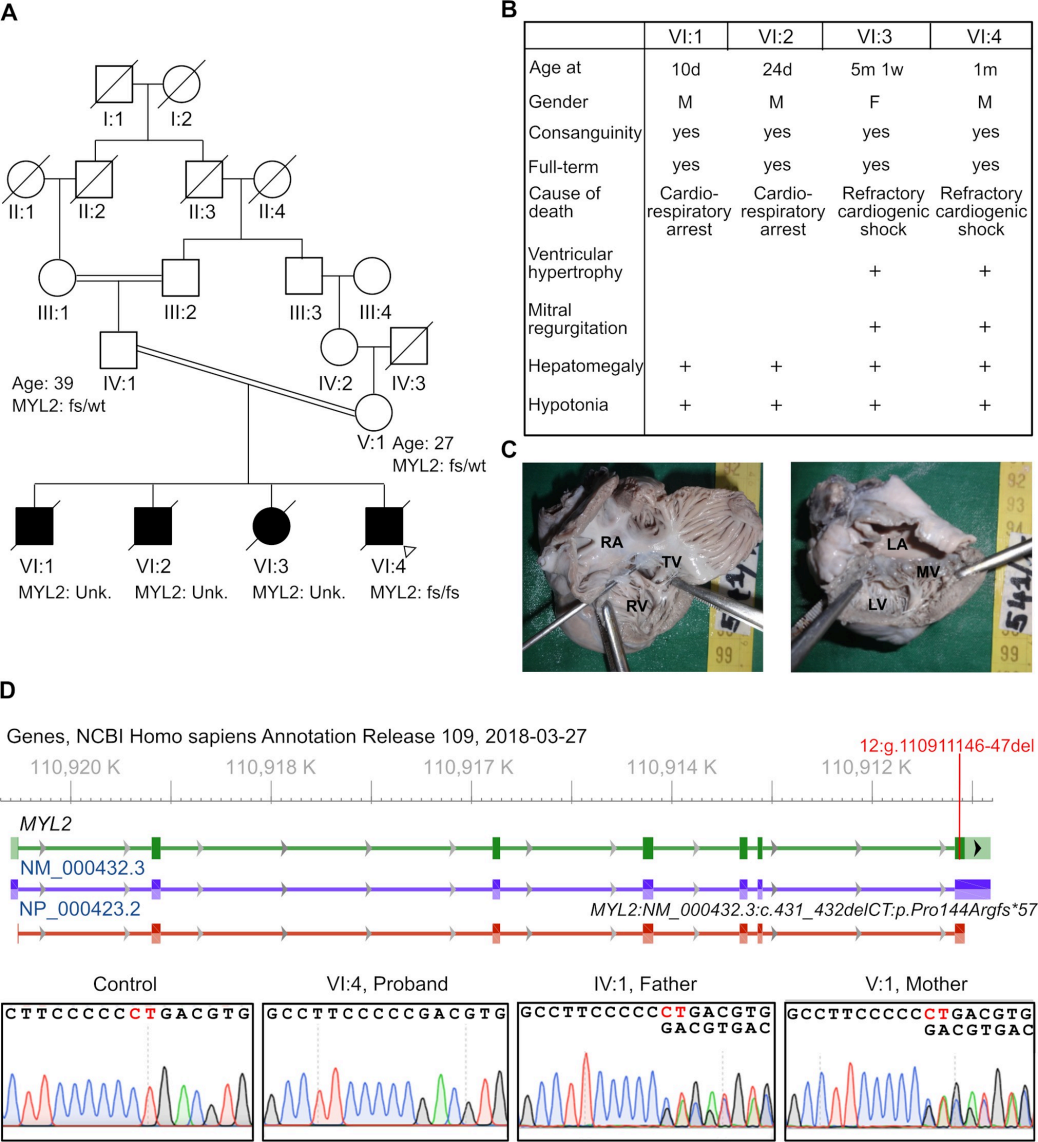
Identification of a novel homozygous frameshift variant in a proband with infantile hypertrophic cardiomyopathy. (A) Pedigree of the family with multiple infant deaths due to early-onset cardiac disease. The consanguinity and age of the parents are also shown. The proband (fs/fs) was homozygous for the *MYL2*-fs allele while the parents were found to be heterozygous (fs/wt) carriers. The status of the *MYL2* allele in siblings is unknown (Unk.). Proband is marked by an arrowhead. (B) Table summarizing key clinical findings of the four siblings (IV:1 –IV:4). (C) Dissection of the post-mortem heart from the proband shows severe biatrial dilatation along with hypertrophy of the right ventricle (left). Severe mitral valve dysplasia and hypertrophy of the left ventricle with a small ventricular cavity is also noted (right). RA- right atrium; TV- tricuspid valve; RV- right ventricle; LA- left atrium; MV- mitral valve; LV- left ventricle (D-top) The genomic locus of the *MYL2* gene shows the frameshift variant identified in the proband (highlighted in red), which is located in the last exon of the gene. (D-bottom) Sequence chromatograms from Sanger sequencing of control (*MYL2 wt*), the proband (homozygous fs variant: *MYL2*:*NM_000432*.*3*:*c*.*431_432delCT*) and the parents (heterozygous fs).

We identified a family with consanguinity in which four children had died before one year of age (Fig 1A). All four children were born at full-term through a Caesarian section and showed no gross developmental defects (Fig 1B). All four showed a rapid decline in health and were treated for various periods in neonatal intensive care units. Two infants (VI:1 and VI:2) died at less than 30 days of age from cardiorespiratory arrest, and two (VI:3 and VI:4, proband) of them displayed abnormal cardiac findings on chest roentgenogram. While all four children displayed hepatomegaly and general hypotonia, VI:3 and VI:4 had a marked increase in heart size and died from refractory cardiogenic shock (Fig 1B, S1A Fig). Echocardiographic evaluation of the proband showed severe biatrial dilatation and biventricular hypertrophy (S1A Fig). The patient also displayed severe mitral valve regurgitation with abnormal thickening of the mitral valve leaflets and pulmonary arterial hypertension. Post-mortem examination of the proband’s heart confirmed severe biatrial dilatation, significant biventricular hypertrophy with small ventricular cavities and severe mitral valve dysplasia (Fig 1C). The father (37 years old) and mother (27 years old) of the proband are consanguineously related (Fig 1A), were asymptomatic from a cardiac standpoint and had normal cardiac anatomy and function as evaluated by an echocardiogram. The similarities in the symptoms between the siblings indicated an underlying genetic cause for the disorder, and we decided to perform genomic analysis to identify potential disease-contributing variants.

### Whole exome sequencing to identify variants and prioritization of variants for functional analysis

We performed exome sequencing on the proband, the mother and the father. Sequencing data were analyzed using our previously published pipeline, Churchill, for calling variants [63]. Variants were prioritized using minor allele frequency (<0.001) and damaging effect prediction by five out of the seven algorithms to filter variants (S1B Fig) [27–32]. This approach resulted in the identification of one *de novo* heterozygous variant in *Olfactory Receptor Family 7 Subfamily C Member 1* (*OR7C1*:NM_198944.1:c.335delA:p.Asn112fs) and a homozygous variant in *Myosin light chain 2* (*MYL2*:NM_000432.3:c.431_432delCT:p.Pro144Argfs*57) (Fig 1D). *OR7C1* encodes an olfactory receptor protein that is not expressed in the heart. Therefore, we focused on the *MYL2* variant (*MYL2-fs*). We confirmed the presence of the homozygous variant in the proband and that the parents are heterozygous carriers using Sanger DNA sequencing of the genomic region (Fig 1D). The dinucleotide deletion in the last exon of *MYL2* is predicted to cause a frameshift mutation that affects the last 20 amino acids in the C-terminal EF-Hand domain. Moreover, the variant extends the reading frame of *MYL2* into the 3′ UTR, leading to the addition of 36 amino acids to the C-terminal end. This *MYL2-fs* variant was not found in control populations in gnomAD, supporting the possibility of its association with this rare disorder.

### Myocyte disarray, fibrosis, and reduction in MYL2 expression in the ventricular muscle of the proband

**Fig 2:**
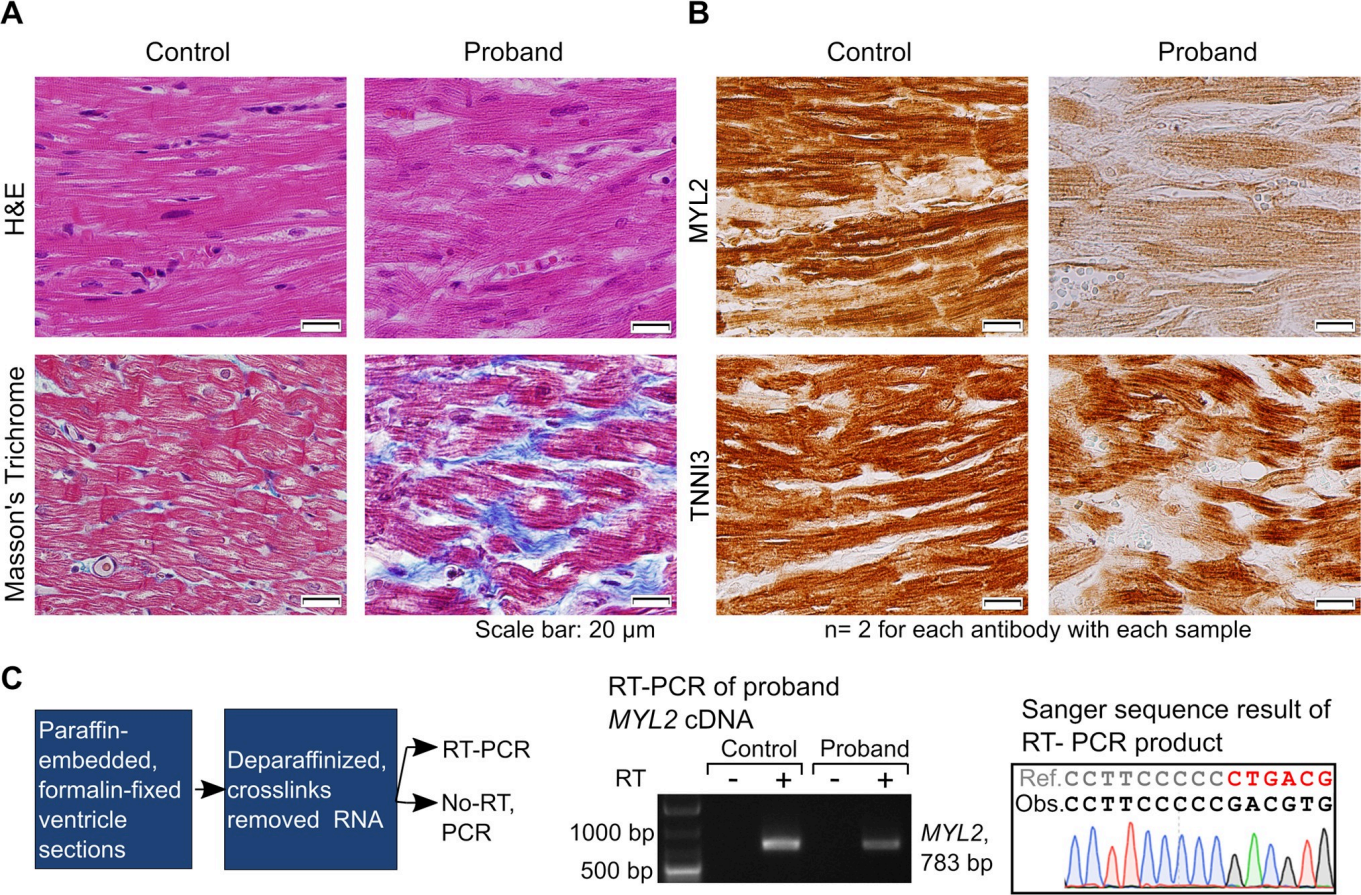
Analysis of the ventricular myocardium of the proband shows characteristic features of hypertrophic cardiomyopathy (HCM) and molecular consequences of the variant. (A) Histochemical analysis of the ventricular myocardium from the proband by H&E staining shows myocyte disarray as compared to control. Masson’s trichrome staining of the proband’s ventricular myocardium shows increased fibrosis (stained with blue). (B) Immunohistochemical analysis of MYL2 expression in the proband’s ventricular myocardium shows a remarkable reduction in the expression of MYL2 as compared to control. The antibody used targets residues at the N-terminal region of the protein (S4 Fig). Cardiac troponin I (TNNI3) was detected at comparable levels in the control and the proband myocardium. The unstained areas correspond to fibrosis. (C) Flow diagram showing the steps used to analyze *MYL2* mRNA expression in the ventricular myocardium of the proband. An RT-PCR product of the expected size of *MYL2* mRNA is detected in the control and proband tissue, and not in the negative (No-RT) controls. Sanger sequencing of the product from the proband shows the *MYL2* mRNA transcript with the dinucleotide deletion.

To test the consequence of the variant, we performed histological evaluation of the ventricular myocardium of the proband. Consistent with HCM diagnosis, the patient's ventricular muscle displayed myocyte disarray and a substantial increase in fibrotic tissue (Fig 2A). Next, we examined the expression level of MYL2 using immunohistochemistry. We observed a marked reduction in MYL2 protein levels in the proband’s ventricular muscle compared to an unaffected control sample but visibly higher than the background (Fig 2B and S2A Fig). In contrast, the expression level of the sarcomeric protein, cardiac troponin I (TNNI3), in the proband was similar to control (Fig 2B). Also, *MYL2* mRNA was detected in the ventricle of the proband by RT-PCR (Fig 2C). This suggests that the frameshift variant adversely affects the levels of MYL2 protein in the proband.

### *In vitro* testing of MYL2 variant protein stability

**Fig 3:**
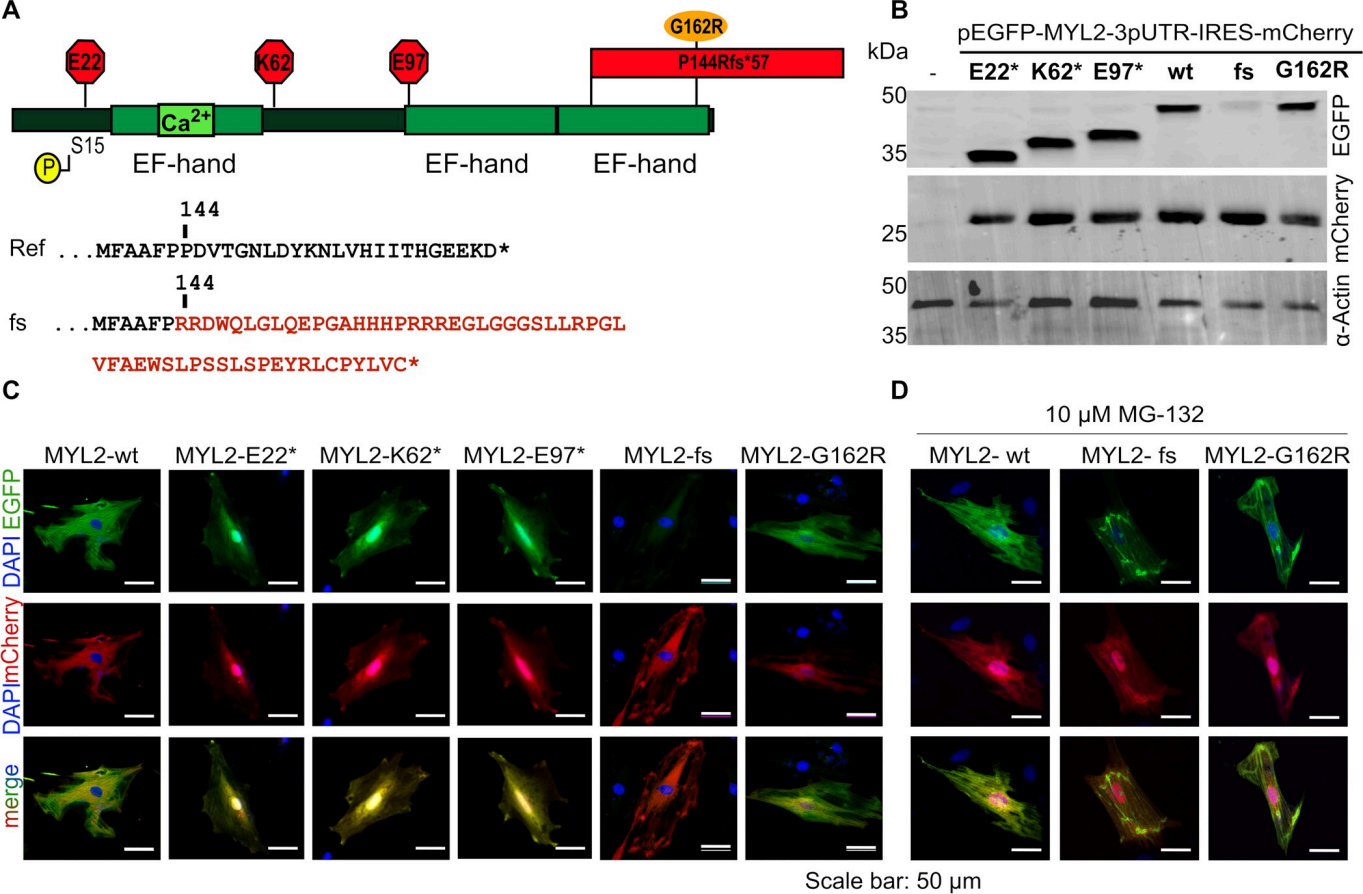
*In vitro* analysis of stability and localization of MYL2 variants. (A) Schematic of MYL2 protein showing N-terminal and C-terminal EF-hand domains along with Serine 15 residue that is target of MLCK phosphorylation, three stop-gain variants (red octagons), the frameshift variant identified in the proband (red block showing extension of C-terminal end) and missense variant associated with HCM (orange oval) in the C-terminal domain. The frameshift mutation changes the canonical MYL2 protein sequence (‘Ref.’, black text) from residue 144 onwards, modifying the last 20 amino acids of the protein in addition to adding 36 non-canonical amino acids to the C-terminus (‘fs’, red text). (B) Western blot image showing overexpression of EGFP-tagged MYL2 wildtype (wt) and variant proteins. The expression of the protein harboring the frameshift variant is significantly reduced while the other stop-gain variants do not show a significant change in expression. Analysis of mCherry signal by western blot does not show any significant changes between MYL2 wt and tested variants. Loading controls: mCherry and Actin. (C) In H9c2 cells, immunofluorescence analysis of EGFP tagged MYL2 wt and tested variants (green) that overexpress mCherry from the same transcript (red) is shown. EGFP-tagged MYL2-wt localizes to the cytoskeleton, while stop-gain variants (E22*, K62*, E97*) do not localize to the cytoskeleton and are observed in a diffuse pattern. EGFP signal from the frameshift variant (fs) is significantly reduced. Localization of the missense (G162R) variant is in a pattern similar to the wt. mCherry signal from the transfected cells does not change between variants. (D) Immunofluorescence images of EGFP-tagged MYL2-wt, MYL2-fs, and MYL2-G162R in MG-132 treated H9c2 cells show the rescue of MYL2-fs variant signal following MG-132 treatment (western blot of the rescue shown in S3B Fig). Scale bar indicates 50 μm length. Quantitation of signal from the western and immunofluorescence experiments is provided in S3B Fig.

We hypothesized that the reduction in the levels of MYL2 in the ventricular myocardium of the proband is due to the instability of the protein product with frameshift mutation. To test this hypothesis, we compared the stability of the wildtype and frameshift mutation overexpressed in rat cardiomyoblast cells (H9c2 cells) [64]. We generated an EGFP-tagged human *MYL2* cDNA construct that also expresses mCherry, permitting simultaneous evaluation of mRNA stability and protein stability (S3B Fig). Using this construct, we tested the stability of the MYL2-frameshift(fs) variant. Also, we tested 4 variants reported in ClinVar: three other stop-gain variants (MYL2:p.E22*, MYL2:p.K62* and MYL2:p.E97*) and a missense variant (MYL2:p.G162R) that mapped to the most C-terminal EF-hand domain (Fig 3A). The stop-gain variants are currently designated as variants of unknown significance in ClinVar even though they are predicted to delete the critical EF-hand domains. While it is likely that the stop-gain variants (not found in the last exon of *MYL2*) will be degraded through nonsense-mediated decay (NMD) when expressed from the genomic loci, the cDNA overexpression analysis allowed us to examine the effect of loss of different domains of the MYL2 protein. Using immunoblot analysis, we observed that overexpression of the MYL2-fs variant was significantly reduced compared to stop-gain variants and the missense variant (Fig 3B, S3B Fig). However, there was no significant difference in the mCherry signal between these constructs (Fig 3B). We noticed that stop-gain variants did not localize in a pattern similar to the wild type MYL2 protein, which showed strong localization along the cell cytoskeleton (Fig 3C). The fs variant, however, was not detected in H9c2 cells compared to the wildtype control. Once again, there was no significant difference in the production of mCherry from fs variant construct, suggesting that the transfection and transcription of these constructs are comparable to the wildtype MYL2 construct (Fig 3C). This prompted us to examine the mode of degradation of the MYL2-fs variant. We examined if the MYL2-fs variant is degraded by the proteasome machinery by treating the cells with MG-132, a commonly used pharmacological inhibitor of the proteasome [65]. Indeed, when the H9c2 cells were treated with MG-132 after transfection with the MYL2-fs construct, the GFP signal corresponding to MYL2-fs was recovered (Fig 3D, S3C Fig). However, it is worth noting that the MYL2-fs variant appears to be aggregating and has retained some affinity towards the cytoskeleton (Fig 3D, S3D Fig). The binding of the MYL2-fs to myosin head may be affected as the modified residues are found close to the lever arm (S2B Fig). The aggregation suggests that the instability of the MYL2-fs variant might be due to misfolding or changes in the biochemical properties of the protein. Together, the *in vitro* experiments indicate that the MYL2-fs variant is not stable and that proteasome-mediated degradation of the translated product plays a key role in the instability of the protein.

### *In vivo* functional analysis using a *Drosophila* model of Myosin light chain knockdown in the dorsal tube

**Fig 4:**
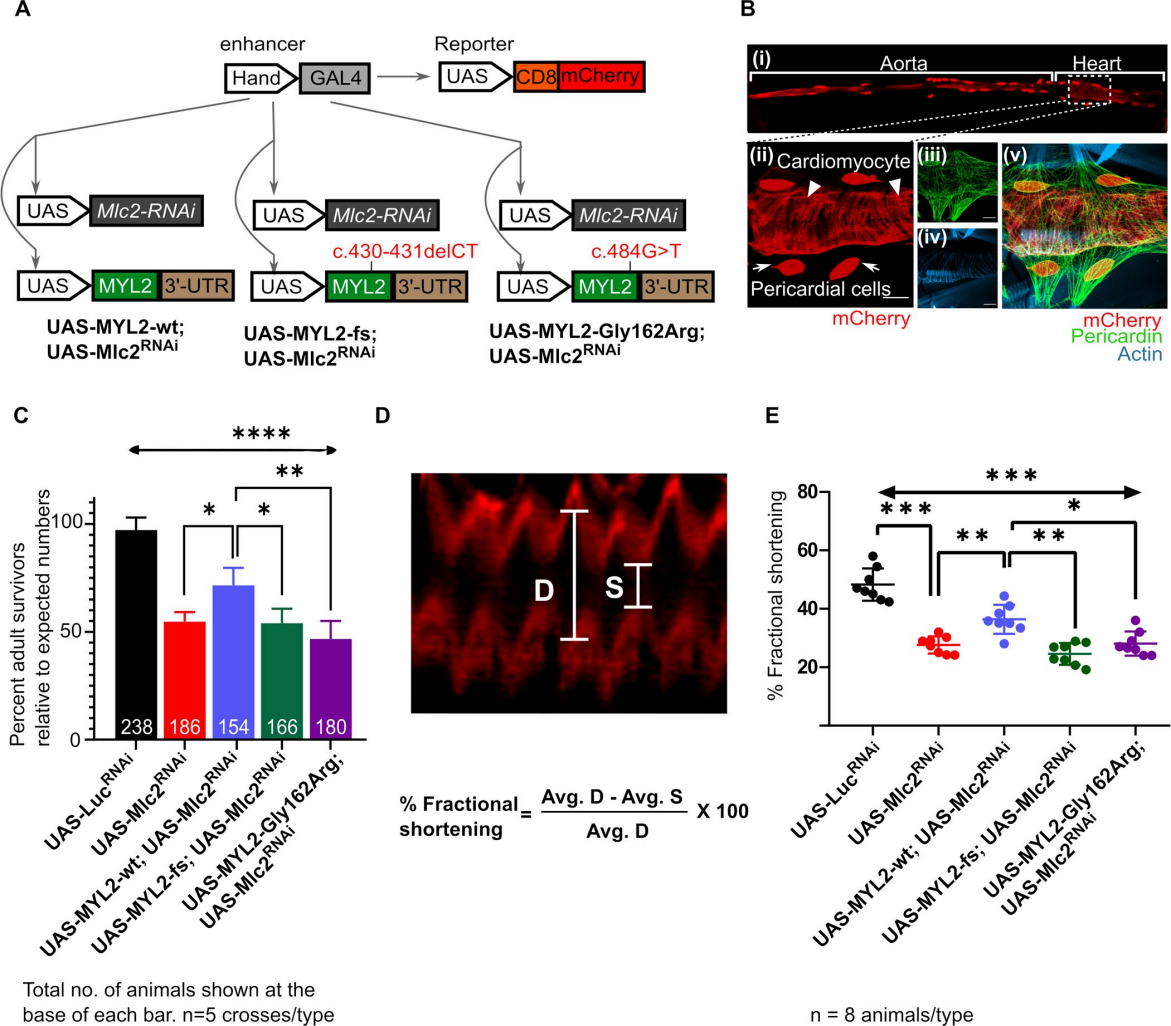
*In vivo* functional analysis of *MYL2* variants using *Drosophila Mlc2* substitution assay in the heart. (A) Schematic shows Hand-enhancer driven *GAL4* (*Hand-GAL4*) that was used to knockdown *Mlc2* expression by RNAi in the heart and to overexpress human MYL2-wt and variant cDNAs for functional substitution. In parallel, the Hand-GAL4 drives the expression of UAS-CD8.mCherry, which facilitates the visualization of the heart. (B-i) Immunofluorescence image of the *Drosophila* heart from UAS-CD8.mCherry. Hand-GAL4 animals show expression of mCherry in the heart chambers and aorta. (B-ii) Magnified image of the A7-A8 posterior denticle band region showing expression of mCherry in both cardiomyocytes (arrowheads) as well as pericardial cells (arrows). The expression of pericardin is shown (green, B-iii). Actin expression (blue, B-iv) shows neighboring skeletal muscles (actin is stained with phalloidin). (B-v) Merged image of the three channels. No expression of mCherry was detected in the skeletal muscle. (C) Graph showing percent of progeny that emerge as adults compared to expected numbers following *Mlc2* RNAi knockdown using Hand-GAL4, and rescue by overexpression of human MYL2-wildtype (wt) and variants. Partial rescue of the lethality is observed with wt *MYL2* cDNA overexpression while no significant difference is observed for the tested variants. Significance is analyzed using Brown-Forsythe and Welch ANOVA across all samples followed by multiple comparisons using Games-Howell multiple comparison test. Significance is indicated using * adj. P-Value < 0.05, ** adj. P-Value < 0.01, **** adj. P-Value < 0.0001. (D) Image showing the rhythmic pattern of cardiac contraction that was observed using UAS-CD8-mCherry reporter driven by Hand-GAL4. Diastolic width is shown by ‘D’, and systolic width by ‘S’. The formula used to calculate fractional shortening is shown. (E) Interleaved scatter plot showing fractional shortening across different genotypes (S1–S5 Movies). Fractional shortening was significantly reduced due to *Mlc2* RNAi knockdown which is partially rescued by overexpression of wt human *MYL2*. The missense and fs variants showed no significant improvement in fractional shortening compared to the wt. Significance was calculated using two-way ANOVA followed by Tukey’s multiple comparison tests. Multiplicity adjusted P-Values of Tukey’s comparison are indicated as * adj. P-Value < 0.05, ** adj. P-Value < 0.01, *** adj. P-Value < 0.001, **** adj.P-Value < 0.0001.

To test if any residual expression of the MYL2-fs variant could support myosin light chain function, we examined the ability of human wildtype *MYL2*, MYL2-fs and MYL2-G162R to rescue the loss of *Drosophila* myosin light chain (*Mlc2;* S4 Fig) expressed in the heart. We used the *Drosophila Hand-GAL4* driver to knock down the expression of *Drosophila Mlc2* in the heart using transgenic RNAi lines (Fig 4A). This led to a significant loss in the developmental viability of progeny expressing the RNAi and/or the transgene (Fig 4C). In the knockdown background, using the same *Hand-Gal4* driver, we overexpressed the human wildtype *MYL2*, MYL2-fs, and MYL2-G162R cDNAs to test their ability to functionally substitute fly *Mlc2* (Fig 4A). We observed that the developmental lethality caused by the *Hand-GAL4* driven knockdown of *Mlc2* was partially rescued by the wildtype human *MYL2* overexpression, while the overexpression of the fs variant identified in our proband or the MYL2-G162R missense variant failed to rescue the phenotype. To examine the specific effect of the knockdown and the variant overexpression in the heart, we focused on the cardiac function in the third instar larvae during the fly development. We used a membranous mCherry reporter (CD8-mCherry) to track the rhythmic contraction of the *Drosophila* heart between the posterior denticle belts (A7-A8) (Fig 4B, S1 Movie). Using this reporter driven by the *Hand-GAL4*, we measured fractional shortening of the heart and compared it between different genotypes (Fig 4D, S1–S5 Movies). We observed that loss of *Mlc2* due to RNAi-mediated knockdown led to a decrease in fractional shortening, which was again partially rescued by the wildtype human *MYL2* cDNA overexpression (Fig 4E). As seen with the developmental lethality, there was no rescue observed with the MYL2-fs or the MYL2-G162R variant in terms of fractional shortening (Fig 4E, S1–S5 Movies). Partial rescue in both experiments is likely attributed to sequence differences between the human and *Drosophil*a homologs. *Drosophila* Mlc2 N-terminal region has additional sequences that are needed for the *Mlc2* function [66]. Nevertheless, the *in vivo* analyses suggest that the fs variant and missense variant are functionally different from the wildtype human *MYL2*, and therefore will not support adequate cardiac function.

## Discussion

Using next-generation exome sequencing methodology and our variant prioritization pipeline, we have identified a rare, novel, and homozygous *MYL2-fs* variant (p.Pro144Argfs*57) in a family with multiple infant deaths and early onset HCM. Consistent with the diagnosis, we found that the patient's ventricular muscle shows a marked increase in fibrosis and myocyte disarray. This homozygous frameshift variant was associated with a significant reduction in MYL2 protein in the ventricular myocardium. Our data support a post-translational mechanism for this reduced expression of MYL2 protein as MYL2 mRNA was detected in the patient’s ventricular muscle and *in vitro* stability testing in H9c2 cells demonstrated that the proteasome pathway plays a role in the mutant protein’s instability. The *in vivo* functional testing further demonstrated that any residual MYL2-fs variant protein will not be able to adequately support cardiac function. While the *in vitro* and *in vivo* tools used in this study were generated to assist in the interpretation of genomic results from a single family with novel *MYL2* variant, they will permit rapid analysis of functional differences in other variants of unknown significance in *MYL2*.

These functional testing results shed light on the molecular pathology of this novel *MYL2-fs* variant as well as reported compound heterozygous and homozygous recessive *MYL2* variants leading to early-onset cardiomyopathy [62, 67]. The instability of the MYL2-fs variant protein suggests that any negative effects of MYL2-fs variant on the myosin function is not likely to be dominant. This conclusion is also supported by several loss-of-function variants reported in gnomAD, rendering the probable likelihood of intolerance for *MYL2* to be zero [68]. The three tested stop-gain variants (MYL2:p.E22*, MYL2:p.K62* and MYL2:p.E97*), which are currently reported as variants of unknown significance associated with HCM in ClinVar, are also likely to be pathogenic only as recessive alleles (either as compound heterozygous or homozygous recessive alleles) and not likely to be dominant due to their expression and localization. This brings to the fore two aspects of MYL2 loss-of-function alleles: 1) Loss-of-function alleles may not contribute to HCM due to haploinsufficiency. 2) There is a strong need to test individual variants to understand their mode of pathogenicity to develop personalized therapeutics. It should be noted that while our data support the conclusion that the homozygosity of *MYL2-fs* variant is contributing to the cardiac disease and lethality found in patient VI:4, we are unable to comment upon if the same genetic etiology is resulting in the early lethality of the siblings (VI:1, VI:2, VI:3). We hypothesize based upon the abnormal clinical cardiac findings that VI:3 likely has a similar genetic mutation but we observe that VI:1 and VI:2 died at 10 and 24 days of age, respectively. As they are products of a consanguineous relationship, the etiology for this early lethality in VI:1 and VI:2 may be due to another genetic cause.

Our work suggests that the morphological development of the ventricular myocardium occurred in the absence of MYL2 protein in the patient with the homozygous *MYL2-fs* variant (p.Pro144Argfs*57). This might be due to the increase in expression of atrial light chain *MYL7* in the embryonic ventricle, as was observed in the mouse model [56]. Whether such a response exists in dominant *MYL2*-missense variant associated myopathies remains to be investigated. This is important due to the observation that pathogenic missense variants display reduced capacity to support myosin function *in vitro* [69]. However, these pathogenic missense variants can still impart a negative effect on myosin function by affecting myosin contractility [48]. This may not be in the case with stop-gain mutants that are likely to be degraded through NMD or frameshift variants that result in a destabilized protein. The observation that loss-of-function alleles display recessive inheritance while missense variants display dominant inheritance in HCM is supported by analysis of animal models [56, 57] and is similar to the genetic analysis of other sarcomeric genes [70]. However, the possibility exists that other variants in the patient could modulate the instability of the MYL2-fs variant, such as reduced proteasome function, that could increase the stability of the protein. Therefore, there is a need to identify modifiers and detect variants in these genes in future patients with *MYL2* frameshift variants to determine the risk of HCM inheritance. Unlike the missense variants, where the allele-specific silencing approach may provide a viable solution [71], the treatment and management of the recessive form of HCM will require early diagnosis and potentially involve gene therapy using a functional copy of *MYL2*. While this may not be immediately feasible, the diagnosis and genetic counseling of carriers need to be considered.

## Materials and methods

### Ethics statement

Informed written consent for the genetic studies was obtained from the parents of the proband as per institutional guidelines that were approved by the Ethics Committee of CHU Habib Bougatfa of Bizerte (Protocol number: 3/17).

### DNA isolation and whole-exome sequencing

DNA was isolated using standard procedures. Exome libraries were constructed using the Agilent SureSelectQXT Target Enrichment System for Illumina Multiplexed Sequencing Protocol (Agilent Technologies, CA). DNA libraries were captured with the Agilent Clinical Research Exome Kit. Paired-end 150 base pair reads were generated for exome-enriched libraries sequenced on the Illumina HiSeq 4000 to a targeted depth of 100× coverage.

### Variant identification, prioritization, and confirmation

The primary and secondary variant analysis was performed as described in our previous studies. The analysis of the sequencing data was conducted using the Churchill pipeline [63], in which the data was aligned to GRCh37 using BWA mem, deduplicated using samblaster, and variants were jointly called across all samples using GATK’s HaplotypeCaller. SnpEff, a software tool to annotate genetic variation, was used along with custom in-house scripts to provide mutation and gene information, protein functional predictions and population allele frequencies. Common variation occurring at >0.1% minor allele frequency in the population was excluded. Variants outside of coding regions (defined as >4 base pairs from an exon splice site) and exonic variants coding for synonymous single nucleotide polymorphisms were also dropped. *In silico* analysis was performed using algorithms to predict the pathogenicity of identified sequence variants. The following prediction software was used to analyze the rare variants in candidate CHD genes identified through WES: SIFT, GERP++, Polyphen2 Complex, Polyphen2 Mendelian, MetaSVM, MetaLR, and CADD [27–32]. Variants were further filtered based on the expression of the impacted human gene in the developing heart using publicly available single-cell data [72]. *MYL2* genomic region flanking the identified variant was amplified using MYL2.gen.For and MYL2.gen.Rev primers and the presence of the variant was confirmed using Sanger sequencing method.

### Total RNA isolation from patient ventricle and RT-PCR

For the control human RNA, 2 mg of frozen ventricular tissue was ground in Trizol (Ambion), and RNA isolated using Total RNA purification plus kit (Norgen Biotek). For the proband, four 7-micron sections of the left ventricle were used to isolate total RNA using the Quick-RNA^TM^ FFPE kit (Zymo Research). 1 μg of total RNA was used to generate cDNAs using the SuperScript™ VILO™ cDNA Synthesis Kit. MYL2 cDNA was amplified using the primers FP.MYL2.BglII and RP.MYL2-3p-UTR.XhoI and the PCR product was sequenced to confirm the expression of MYL2-fs variant.

### Plasmid constructions

Human *MYL2* cDNA (Refseq ID NM_000432.3) in the pDNR-LIB plasmid was obtained from Harvard Plasmid Database. *MYL2* coding region with the 3′ UTR sequence was amplified using PCR with primers FP.MYL2.BglII and RP.MYL2.EcoRI, and cloned into *Bgl*II/*Eco*RI site in the pIRES-mCherry plasmid (a gift from Ellen Rothenberg (Addgene plasmid # 80139; http://n2t.net/addgene:80139; RRID: Addgene_80139). Then, *MYL2*-coding region, 3′ UTR sequence and IRES mCherry were excised using *Bgl*II/*Cla*I and introduced in frame with the EGFP tag in a modified pEGFP-C1 plasmid (Clonetech) to generate the pEGFP-MYL2-IRES-mCherry plasmid. This construct, when transfected in cells, leads to the production of an EGFP tagged MYL2 protein (or MYL2 variant protein) and independently translated mCherry from the same transcript. The EGFP tag distinguishes the overexpressed cDNA from rat *Myl2*, while the independently produced mCherry acts as a readout of mRNA transcription and also serves as a transfection control allowing direct comparison of cellular levels of the overexpressed MYL2 protein variants. For *Drosophila* transgenic experiments, the untagged MYL2 coding region with the 3′-UTR was amplified using FP.MYL2.BglII and RP.MYL2-3p-UTR.XhoI inserted using *Bgl*II/*Xho*I in pUASt-attB-exp (a modified pUASt-attB vector with additional restriction sites) to generate pUASt-MYL2-attB. To generate the variants, site-directed mutagenesis was used with pEGFP-MYL2-IRES-mCherry or pUASt-MYL2-attB as the template and using the following primers and Agilent Quickchange II kit [73]. Sequences are provided in S1 Table.

MYL2-fs (c.431-432delCT): FP.delCT-431-432.MYL2, RP.delCT-431-432.MYL2

MYL2-G162R: FP.MYL2.G162R, RP.MYL2.G162R

MYL2-E22*: RP.MYL2E22Stop, FP.MYL2E22Stop

MYL2-K62*: FP.MYL2K62Stop, RP.MYL2K62Stop

MYL2-E97*: RP.MYL2E97Stop, FP.MYL2E97Stop

### Cell culture

H9c2 cells [64] were cultured in Dulbecco's Modified Eagle's Medium (DMEM) with 4.5 g/L Glucose, 4 mM L-Glutamine, 1 mM sodium pyruvate, and 1.5 g/L sodium bicarbonate (ATCC 30–2002), supplemented with 10% fetal bovine serum, 100 I.U./mL penicillin and 100 (μg/mL) streptomycin at 37°C incubator with 5% CO_2_. Cells were transfected with 2 μg of the plasmid with Lipofectamine 3000 reagent with OptiMEM media according to manufacturer's recommendations. Transfection media were removed five hours post-transfection and replaced by normal growth media. Cells were collected for Immunoblot analysis or Immunofluorescence 48 hours after transfection. For proteasome inhibition, 24 hours post-transfection, cells were treated with 10 μM MG-132 in growth media and incubated for another 24 hours before analysis.

### Fly stocks

*Drosophila* lines were maintained in standard fly food with yeast at 25°C. The following stocks were obtained from the Bloomington *Drosophila* Stock Center: *UAS-CD8*.*mCherry* (BDSC 27391), *Hand-enhancer-GAL4* (BDSC 48396), ‘Dm integrase with attP landing site VK37’ (BDSC 24872), *Mlc2*^*RNAi*^ (JF01106, BDSC 31544), Luciferase (firefly)^RNAi^ (BDSC 31603), Multiple balancer stock with mCherry marker (BDSC 76237; CyO, P{sqmCh}2; TM3, P{sqmCh}3, Sb), how(24B)-GAL4 (BDSC 1767).

### *Drosophila* transgenesis and crossing schemes

UAS-*MYL2*-attB constructs (300 ng/μL) were injected into ‘Dm integrase with landing site VK37’ stock to generate overexpression transgenic stocks using previously described methods with small modifications. F0 injection-survivors were crossed to *yw* animals and transgenic progenies from this cross (F1) were identified using red eye color. Stocks were generated using standard *Drosophila* mating schemes to generate UAS-MYL2 (wt or variant)/CyO P{sqmCh}2; Mlc2^RNAi^/TM3, Sb, P{sqmCh}3 stocks. These were crossed to UAS-CD8.mCherry/CyO; GMR88D05-Hand GAL4/TM6, Ubx-LacZ animals and raised at 29°C to maximize the effect of GAL4 mediated transcription. For developmental lethality, emerging adults irrespective of sex were used to determine percent adult survivors and compared to control crosses where *Luciferease* RNAi was overexpressed using *Hand-GAL4*. Percent of progeny expressing the RNAi as well as the transgenes (inferred from the absence of phenotypic markers of the balancer chromosomes) that emerge as adults compared to the expected number of flies among siblings (based on the Mendelian ratio) from each of the crosses was used to determine developmental viability. Knock-down of *Mlc2* using *Hand-GAL4* likely leads to multiphasic lethality with animals dying as embryo and larvae before the pupal stage. For cardiac function analysis, larvae of the desired genotype were identified using mCherry signal in the heart and the absence of muscle mCherry signal from the balancer chromosome.

### Fluorescent reporter-based fly cardiogram

Crawling-third instar larvae were collected for each genotype and immobilized on a double-sided tape on a glass slide with the dorsal side of the larvae towards the camera. Videos were collected using an Olympus BX51 microscope with a DP71 camera. Each larva was imaged two times for 15 seconds each with 15 seconds gap. Kymographs of the videos were created, and systolic-diastolic widths of the heart tube were measured using ImageJ across 10 different contractions and averaged between the two videos for each animal. Fractional-shortening was calculated as described in previous studies using the formula: % fractional shortening = (Avg. D–Avg. S) / (Avg. D) *100 where ‘D’ is the diastolic width and ‘S’ is the systolic width [74].

### Western blot

Immunoblots were performed using standard protocols prescribed for the LI-COR biosciences method of detection using infra-red dye conjugated secondary antibodies. The following antibodies were used: Chicken anti-GFP (1:1000; Abcam: ab13970), Rabbit anti-mCherry (1:1000; Abcam: ab167453) and Rabbit anti-actin (1:1000; Abcam: ab1801). Rabbit anti MYL2 (1:1000; Abcam:48003) with HRP-conjugated anti-Rabbit antibody (1:1000; Vector laboratories, PI-1000) was used to evaluate overexpression of human MYL2 wt and variants through western blot of total protein from corresponding *Drosophila* larvae.

### Histology, immunohistochemistry, and immunofluorescence

Formalin-fixed and paraffin-embedded ventricular myocardium of the deceased proband from the Department of Embryo-Fetopathology of Tunis and control heart from an unaffected donor of comparable age obtained from the Heart Center Biorepository at Nationwide Children’s Hospital were sectioned and processed using standard methods. Hematoxylin and Eosin Staining [75] and Masson’s trichrome staining [76] were used to study the histology of the samples. For immunohistochemistry following antibodies were used: Rabbit Anti-Myosin light chain 2 antibodies (1:500, Abcam: ab48003), Rabbit anti cardiac troponin I (TNNI3) (1:500, Abcam ab47003). For immunofluorescence, cells were fixed using 4% paraformaldehyde and processed for immunofluorescence using standard protocols. The following antibodies were used: Chicken anti-GFP (1:500; Abcam: ab13970) and Rabbit anti-mCherry (1:500; Abcam: ab167453). *Drosophila* larvae were dissected and stained with the Pericardin antibody (1:100, Developmental Studies Hybridoma Bank: EC11) as described previously.

## Supporting information

S1 Fig Clinical diagnosis and identification of rare variants in siblings. (A) Radiographs showing hepatomegaly in all four siblings and cardiomegaly in the proband (VI:4) and sister (VI:3). Echocardiogram of the proband showing severe biatrial dilation and small ventricular cavities with significant septal hypertrophy (left). Images are comparable to post-mortem analysis shown in Fig 1C. (B) Flow diagram showing the number of genes with variants passing through each filter in our variant prioritization pipeline. Variants are classified into *de novo*, homozygous and compound heterozygous variants before prioritization. Three filters are used to reduce possible sequencing artifacts (quality score cutoff), common variants (frequency in general population cutoff), and variants predicted to be benign (damaging effect prediction cutoff). One *de novo* and one homozygous variant were prioritized using this pipeline.
(TIF)

S2 Fig Immunohistochemical analysis of MYL2 in the myocardium of the proband and model of Myosin interacting-heads motif (IHM) that shows impacted residues. (A) (i) Image of control ventricular myocardium stained with MYL2 antibody that recognizes the N-terminal region of the protein is shown. (ii) Ventricular myocardium from proband shows weak signal suggesting a marked reduction in MYL2 expression. (iii) Primary antibody negative control shows background signal. (B) A model of the myosin interacting head motif showing myosin heavy chain (blue) and two interacting light chains: essential light chain (red) and regulatory light chain (green-pink). In the regulatory light chain, residues affected by the frameshift variant are shown in pink with the starting residue (Pro144) depicted by spheres. The proximity of the variant residues to the IHM suggests that it can affect the binding of the regulatory light chain to the myosin head.
(TIF)

S3 Fig *MYL2* variants, reporters and in vitro assays. (A) Schematic of MYL2 primary structure that shows domains and missense variants reported in ClinVar (cyan bubbles). The frameshift variant is shown as a red box. (B) EGFP-tagged MYL2 overexpression vectors show various overexpression products that are expected from each construct. (C) Immunoblot against GFP shows the rescue of MYL2-fs variant upon addition of MG-132 to H9c2 cells transfected with the EGFP-tagged overexpression constructs (left). Quantitation of immunoblot based stability analysis of *MYL2* variants and MG-132 mediated rescue of the EGFP-tagged MYL2 signal is shown (center). Immunofluorescence based stability analysis is shown (right). One-way ANOVA was used to test for significance and multiplicity adjusted P-Values from Tukey’s multiple comparisons are shown. ANOVA **** P-Value < 0.0001. (D) Additional panels show multiple fields-of-view(F1-F8) of the rescue of EGFP tagged MYL2-fs signal in H9c2 cells. Cells show various levels of aggregation and limited localization to the cytoskeleton. mCherry signal shows transfected cells.
(TIF)

S4 Fig Conservation of amino acid sequence between *MYL2* homologs and overexpression of human *MYL2* variants in *Drosophila*. (A) MUSCLE alignment of human *MYL2* and homologs from different species show high conservation of residues along the length of the protein. Residues targeted by the antibody against MYL2 (ab48003) are highlighted in the green box. (B) RT-qPCR result shows expression of human *MYL2* mRNA in *held-out wings* (how(24B)-GAL4) driven *UAS-MYL2*. Expression of how(24B)-GAL4 driver is broadly detected in skeletal muscles and cardiomyocytes among other tissues [77]. Western blot analysis MYL2 of protein from larvae overexpressing human *MYL2* (wt or variant) transgene under the control of *how(24B)-GAL4*. Wt and G162R (~19 kDa) variants were detected in the total protein lysate, while the fs variant was not detected. *Drosophila* endogenous Mlc2 (23 kDa) is also detected due to the conservation of the antibody target region (highlighted within the green box in A).
(TIF)

S1 Movie Third instar larval heart used as control (GMR-Hand-GAL4> UAS-Luc^RNAi^). (MOV)

S2 Movie Third instar larval heart shows the impact of Mlc2 knockdown (GMR-Hand-GAL4> UAS-Mlc2^RNAi^). (MOV)

S3 Movie Third instar larval heart shows the partial rescue of Mlc2 knockdown by human MYL2 wt cDNA overexpression (GMR-Hand-GAL4> UAS-Mlc2^RNAi^, UAS-MYL2-wt). (MOV)

S4 Movie Third instar larval heart shows no rescue of Mlc2 knockdown by human MYL2-fs cDNA overexpression (GMR-Hand-GAL4> UAS-Mlc2^RNAi^, UAS-MYL2-fs). (MOV)

S5 Movie Third instar larval heart shows no rescue of Mlc2 knockdown by human MYL2-G162R cDNA overexpression (GMR-Hand-GAL4> UAS-Mlc2^RNAi^, UAS-MYL2-G162R). (MOV)

S1 Table Sequences of primers used in the study. (DOCX)
