# Supplementary figures and images for: Novel frameshift variant in MYL2 reveals molecular differences between dominant and recessive forms of hypertrophic cardiomyopathy
Source: PLoS Genet. 2020 May 26;16(5):e1008639. doi: 10.1371/journal.pgen.1008639 (PMC7274480; doi:10.1371/journal.pgen.1008639)

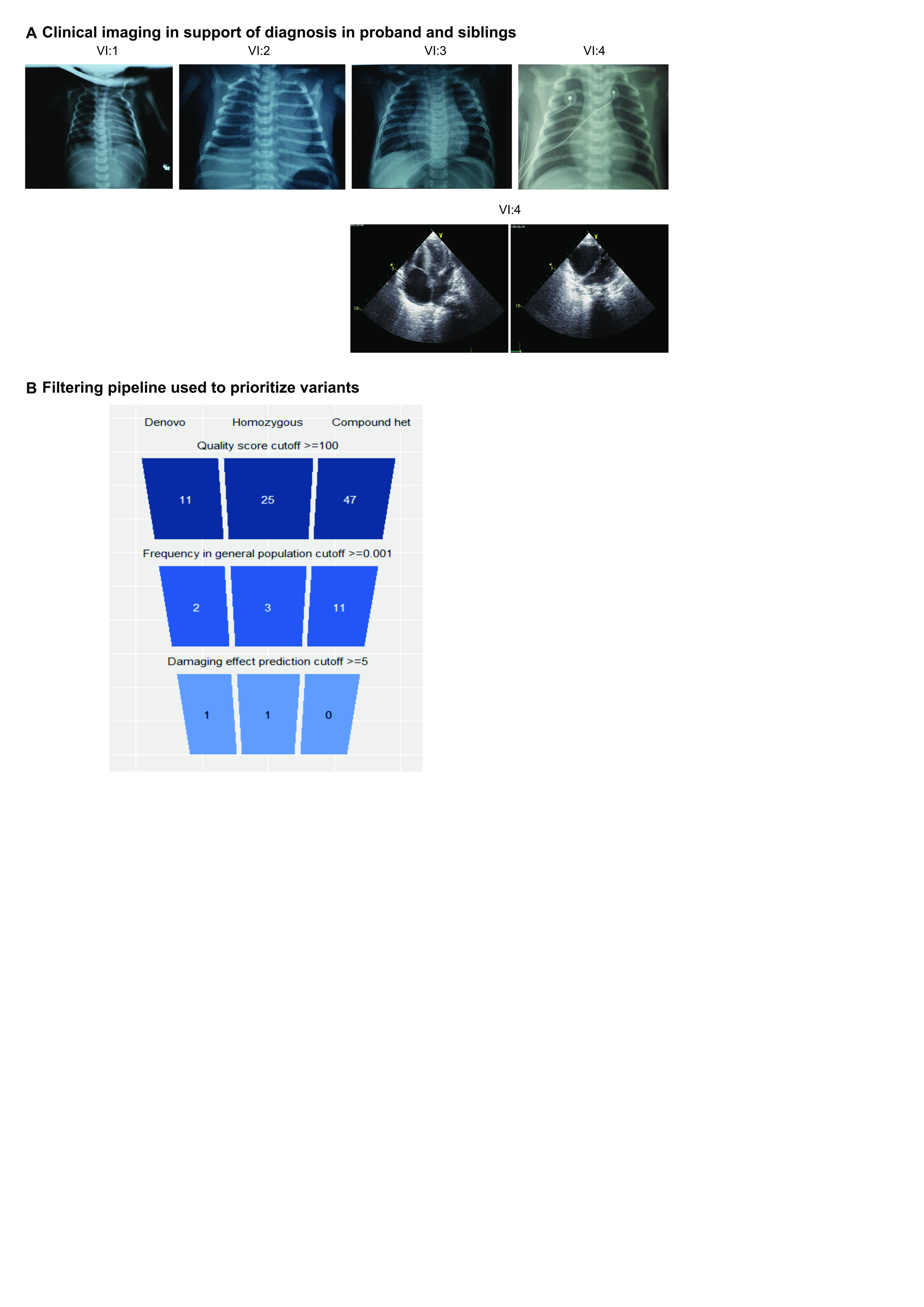

Supplement: S1 Fig — (A) Radiographs showing hepatomegaly in all four siblings and cardiomegaly in the proband (VI:4) and sister (VI:3). Echocardiogram of the proband showing severe biatrial dilation and small ventricular cavities with significant septal hypertrophy (left). Images are comparable to post-mortem analysis shown in Fig 1C. (B) Flow diagram showing the number of genes with variants passing through each filter in our variant prioritization pipeline. Variants are classified into de novo, homozygous and compound heterozygous variants before prioritization. Three filters are used to reduce possible sequencing artifacts (quality score cutoff), common variants (frequency in general population cutoff), and variants predicted to be benign (damaging effect prediction cutoff). One de novo and one homozygous variant were prioritized using this pipeline. (TIF) [file pgen.1008639.s001.tif]

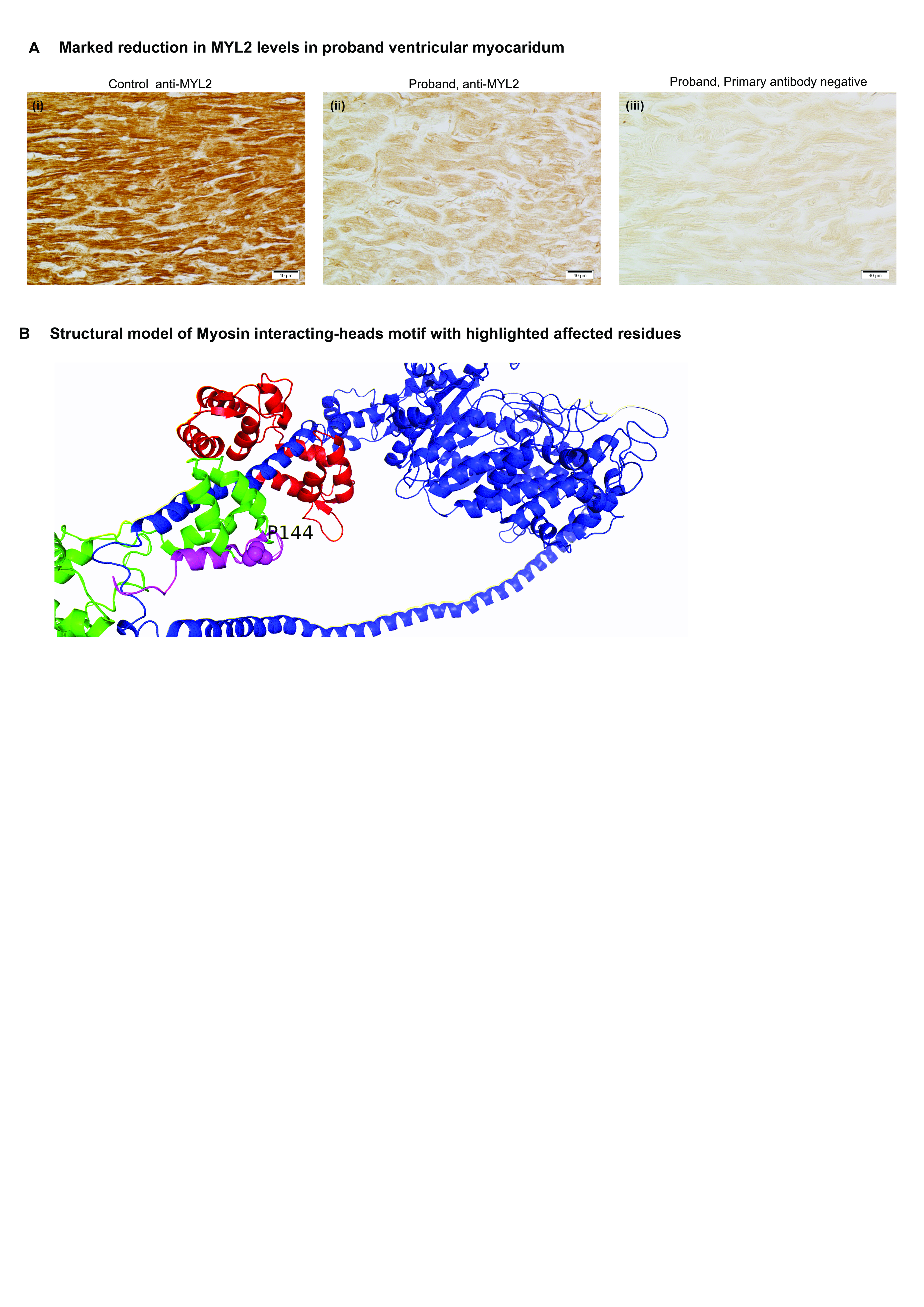

Supplement: S2 Fig — (A) (i) Image of control ventricular myocardium stained with MYL2 antibody that recognizes the N-terminal region of the protein is shown. (ii) Ventricular myocardium from proband shows weak signal suggesting a marked reduction in MYL2 expression. (iii) Primary antibody negative control shows background signal. (B) A model of the myosin interacting head motif showing myosin heavy chain (blue) and two interacting light chains: essential light chain (red) and regulatory light chain (green-pink). In the regulatory light chain, residues affected by the frameshift variant are shown in pink with the starting residue (Pro144) depicted by spheres. The proximity of the variant residues to the IHM suggests that it can affect the binding of the regulatory light chain to the myosin head. (TIF) [file pgen.1008639.s002.tif]

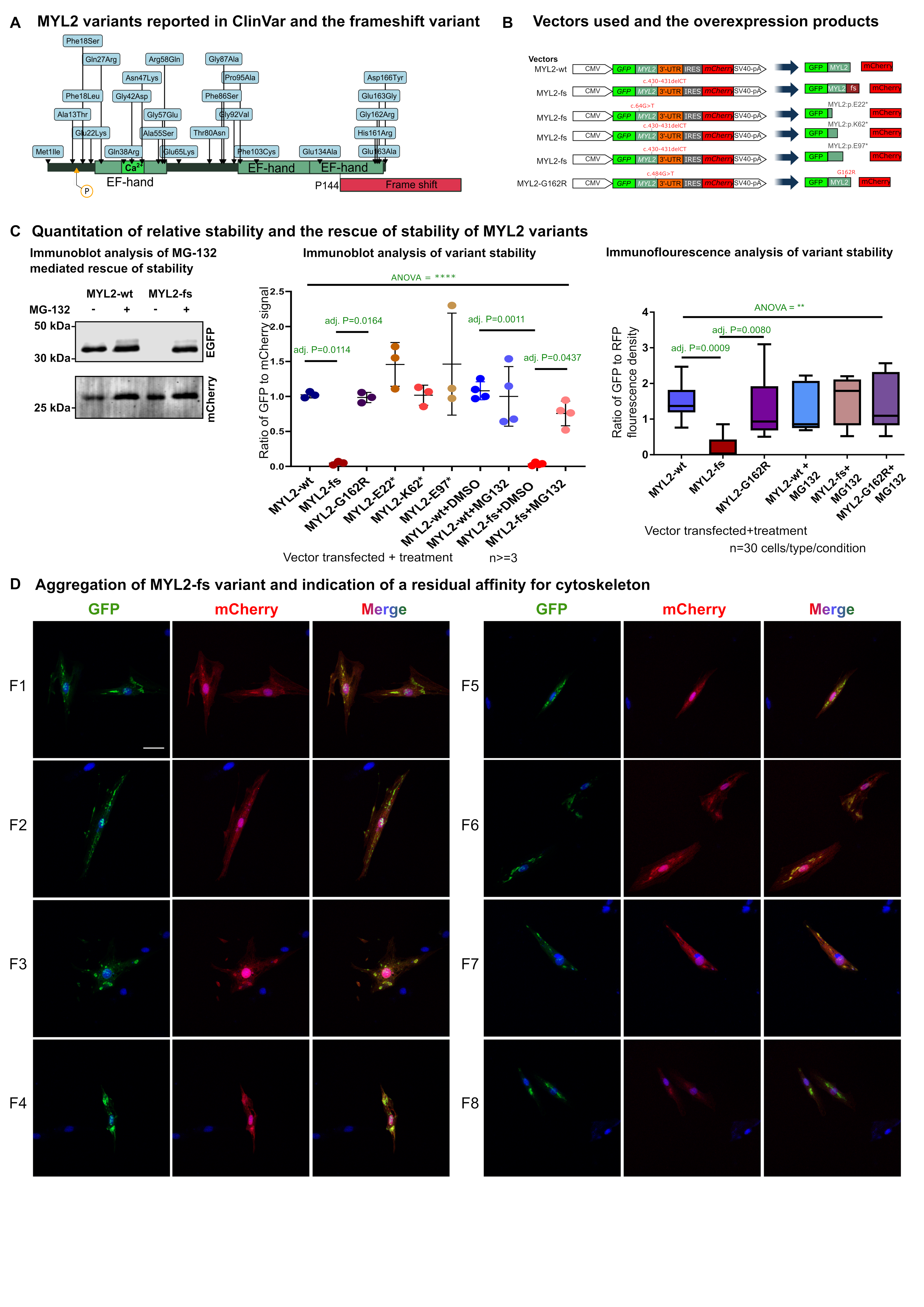

Supplement: S3 Fig — (A) Schematic of MYL2 primary structure that shows domains and missense variants reported in ClinVar (cyan bubbles). The frameshift variant is shown as a red box. (B) EGFP-tagged MYL2 overexpression vectors show various overexpression products that are expected from each construct. (C) Immunoblot against GFP shows the rescue of MYL2-fs variant upon addition of MG-132 to H9c2 cells transfected with the EGFP-tagged overexpression constructs (left). Quantitation of immunoblot based stability analysis of MYL2 variants and MG-132 mediated rescue of the EGFP-tagged MYL2 signal is shown (center). Immunofluorescence based stability analysis is shown (right). One-way ANOVA was used to test for significance and multiplicity adjusted P-Values from Tukey’s multiple comparisons are shown. ANOVA **** P-Value < 0.0001. (D) Additional panels show multiple fields-of-view(F1-F8) of the rescue of EGFP tagged MYL2-fs signal in H9c2 cells. Cells show various levels of aggregation and limited localization to the cytoskeleton. mCherry signal shows transfected cells. (TIF) [file pgen.1008639.s003.tif]

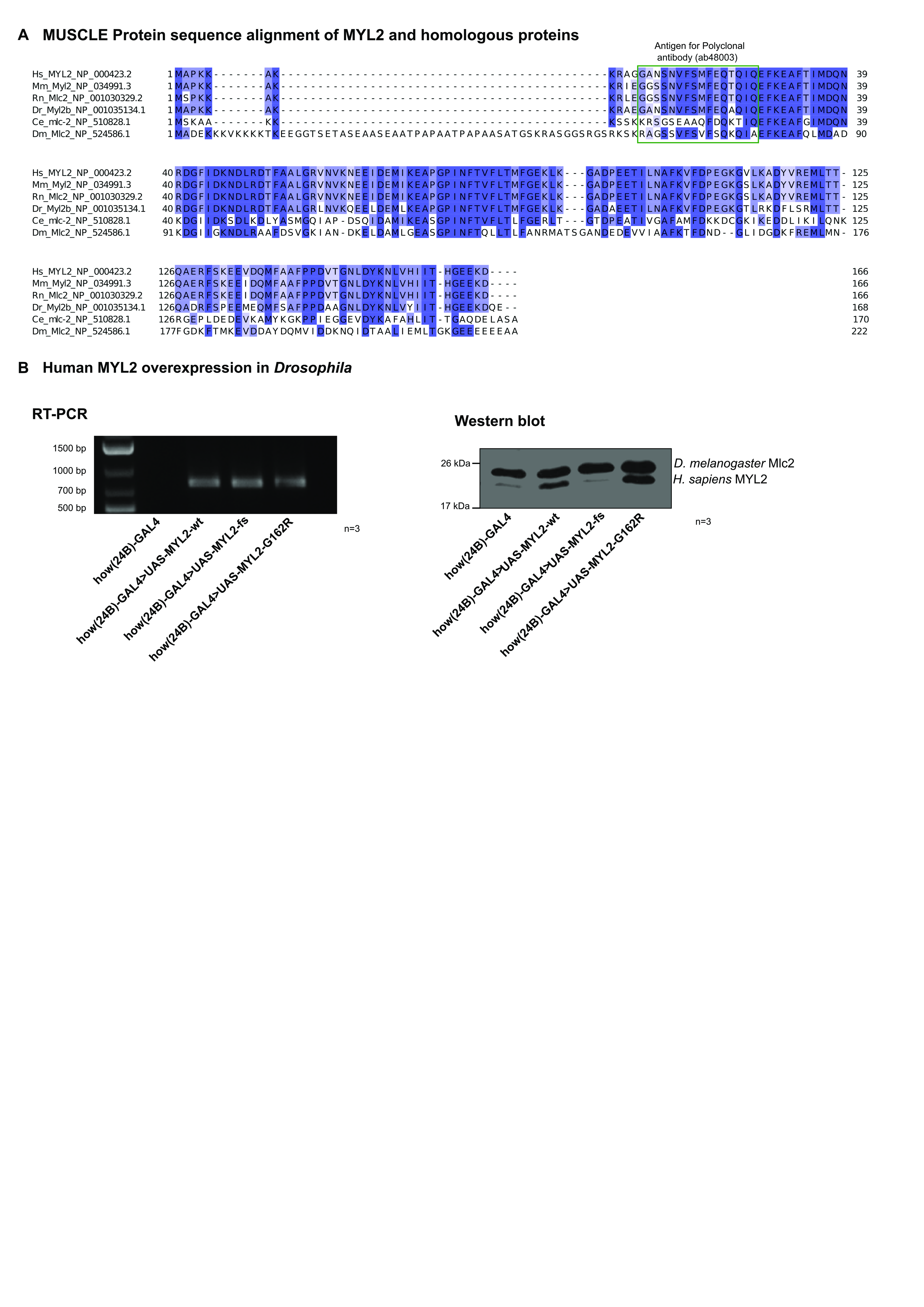

Supplement: S4 Fig — (A) MUSCLE alignment of human MYL2 and homologs from different species show high conservation of residues along the length of the protein. Residues targeted by the antibody against MYL2 (ab48003) are highlighted in the green box. (B) RT-qPCR result shows expression of human MYL2 mRNA in held-out wings (how(24B)-GAL4) driven UAS-MYL2. Expression of how(24B)-GAL4 driver is broadly detected in skeletal muscles and cardiomyocytes among other tissues [77]. Western blot analysis MYL2 of protein from larvae overexpressing human MYL2 (wt or variant) transgene under the control of how(24B)-GAL4. Wt and G162R (~19 kDa) variants were detected in the total protein lysate, while the fs variant was not detected. Drosophila endogenous Mlc2 (23 kDa) is also detected due to the conservation of the antibody target region (highlighted within the green box in A). (TIF) [file pgen.1008639.s004.tif]
