# Supplementary material for: Novel frameshift variant in MYL2 reveals molecular differences between dominant and recessive forms of hypertrophic cardiomyopathy
Source: PLoS Genet. 2020 May 26;16(5):e1008639. doi: 10.1371/journal.pgen.1008639 (PMC7274480; doi:10.1371/journal.pgen.1008639)
Supplement: S1 Table — (DOCX) [file pgen.1008639.s010.docx]

**Table S1: Sequence of primers used in the study**

| **Primer name** | **Sequence (5′-3′)** |
| --- | --- |
| **FP.MYL2.BglII** | aaaAGATCTATGGCACCTAAGAAAGCAAAGAAG |
| **RP.MYL2.EcoRI** | AAAGAATTCGCCAGAGAAATGTCGTGACC |
| **FP.delCT-431-432.MYL2** | GATGTTCGCCGCCTTCCCCCGACGTGACTGGCAACTTGGAC |
| **RP.delCT-431-432.MYL2** | GTCCAAGTTGCCAGTCACGTCGGGGGAAGGCGGCGAACATC |
| **FP.MYL2.G162R** | GGTGCACATCATCACCCACAGAGAAGAGAAGGACTAGGAG |
| **RP.MYL2.G162R** | CTCCTAGTCCTTCTCTTCTCTGTGGGTGATGATGTGCACC |
| **RP.MYL2E22Stop** | CCTGGATTTGGGTCTGTTAGAACATGGAGAACACGTTGG |
| **FP.MYL2E22Stop** | CCAACGTGTTCTCCATGTTCTAACAGACCCAAATCCAGG |
| **RP.MYL2E97Stop** | GAATGCGTTGAGAATGGTTTACTCAGGGTCCGCTCCCTTAAG |
| **FP.MYL2E97Stop** | CTTAAGGGAGCGGACCCTGAGTAAACCATTCTCAACGCATTC |
| **FP.MYL2K62Stop** | GCCCTTGGGCGAGTGAACGTGTAAAATGAAGAAATTGATGAAATG |
| **RP.MYL2K62Stop** | CATTTCATCAATTTCTTCATTTTACACGTTCACTCGCCCAAGGGC |
| **RP.MYL2-3p-UTR.XhoI** | aaaCTCGAGgagggataacttcgtatagcagaattc |
| **MYL2.gen.For** | GTGGCCCATGTTCCCATC |
| **MYL2.gen.Rev** | GGTACTCGGGGGAGAGAGA |
